# Twelve-year global publications on small incision lenticule extraction: A bibliometric analysis

**DOI:** 10.3389/fmed.2022.990657

**Published:** 2022-09-08

**Authors:** Tian Han, Liang Zhao, Yang Shen, Zhi Chen, Dong Yang, Jiaoyan Zhang, Walter Sekundo, Rupal Shah, Jinhui Tian, Xingtao Zhou

**Affiliations:** ^1^Department of Ophthalmology, Eye and ENT Hospital of Fudan University, Shanghai, China; ^2^NHC Key Laboratory of Myopia, Fudan University, Shanghai, China; ^3^Research Center of Ophthalmology and Optometry Shanghai, Shanghai, China; ^4^Shanghai Engineering Research Center of Laser and Autostereoscopic 3D for Vision Care (20DZ2255000), Shanghai, China; ^5^Evidence-Based Medicine Center, School of Basic Medical Sciences, Lanzhou University, Lanzhou, Gansu, China; ^6^Key Laboratory of Evidence-Based Medicine and Knowledge Translation of Gansu Province, Lanzhou, China; ^7^The School of Nursing, Lanzhou University, Lanzhou, Gansu, China; ^8^The Department of Ophthalmology, Philipps University of Marburg, Marburg, Germany; ^9^New Vision Laser Centers, Vadodara, Gujarat, India

**Keywords:** bibliometric analysis, small incision lenticule extraction, SMILE, femtosecond laser technology, complications

## Abstract

**Purpose:**

To analyze the development process of small incision lenticule extraction (SMILE) surgery in a 12-year period.

**Methods:**

We conducted a literature search for SMILE research from 2011 to 2022 using the Science Citation Index Expanded (SCIE) of the Web of Science Core Collection (WoSCC). The VOS viewer, and CiteSpace software were used to perform the bibliometric analysis. Publication language, annual growth trend, countries/regions and institutions, journals, keywords, references, and citation bursts were analyzed.

**Results:**

A total of 731 publications from 2011 to 2022 were retrieved. Annual publication records grew from two to more than 100 during this period. China had the highest number of publications (n = 326). Sixty-five keywords that appeared more than four times were classified into six clusters: femtosecond laser technology, dry eye, biomechanics, visual quality, complications, and hyperopia.

**Conclusion:**

The number of literatures has been growing rapidly in the past 12 years. Our study provides a deep insight into publications on SMILE for researchers and clinicians with bibliometric analysis for the first time.

## Introduction

Small incision lenticule extraction (SMILE) is the newest laser vision correction procedure, where the refractive lenticule cut by a femtosecond laser is extracted through a small corneal incision (1). The basic principle of SMILE surgery is similar to that of traditional corneal refractive surgery, which corrects myopia by changing the corneal curvature. The most creative design of SMILE surgery involves a small incision. Coincidentally, the 2-mm incision at the edge also resembles a smile. SMILE surgery evolved from femtosecond lenticule extraction (FLEx). Thanks to the precise resection obtained by the femtosecond laser technique, FLEx was first introduced at the American Academy of Ophthalmology Annual Meeting in 2006 by Walter Sekundo and Marcus Blum, and was first reported by Walter Sekundo et al. (2). Subsequently, researchers found that a more minimally invasive surgery can be achieved by a small surgical incision. The earliest pieces of literature on SMILE surgery were published by Walter Sekundo et al. and Rupal Shah et al. (3, 4). At present, SMILE surgery has gradually become one of the most widely used corneal refractive surgeries. Like a single spark that could kindle a whole prairie, the evolution of SMILE surgery over the past 12 years has been drastic. Currently, the number of SMILE surgeries has reached six million globally. A large number of studies on this surgery have also been published.

Bibliometrics involve scientific summarization of the literature through intuitive charts, which makes it easy to understand the countries, institutions, authors, journals, and hotspots of related disciplines. This study aimed to analyze the research progress in SMILE surgery over the last 12 years through bibliometrics.

## Materials and methods

### Data source

We conducted a literature search for SMILE research from 2011 to 2022 using the Science Citation Index-Expanded (SCIE) of the Web of Science Core Collection (WoSCC) to identify SMILE-related publications, limited to “article” and “review”, over the past 12 years (from 2011 to 2022) with no language restriction. Our search strategy was as follows: Topic = (“small incision lenticule extraction” OR “small incision lenticule extractions” OR SMILE*). All retrieved records were downloaded on May 14, 2022.

### Statistical analysis

The annual number of publications, type of documents, and languages on SMILE studies were analyzed using CiteSpace 6.2.1 (Drexel University, Philadelphia, PA, United States). The impact factors of the journals were provided by the 2021 Journal Citation Reports (Clarivate Analytics, Philadelphia, PA, United States). Elements of SMILE research, including countries/regions, keywords, journals, and main co-cited journals, were identified via VOS viewer 1.6.15 (Leiden University, Leiden, Netherlands). A publication was assigned equally to all participating countries/regions or institutions when it was completed by collaborations between more than one country/region or institution. Network maps for countries/regions, institutions, journals, and the main co-cited journals were generated by the VOS viewer in addition to cluster analysis and density maps for high-frequency keywords. On the bibliometric maps generated by the VOS viewer, different nodes represent elements, and the larger the size of the node, the higher the number or frequency of elements is. A line, which connects two nodes, reflects the relationship between different elements, and its thickness indicates the strength of the relationship. Nodes of different colors represent different clusters. Parameters of the VOS viewer were set as follows: fractional counting at the counting method, ignoring documents with too many authors (maximum number of authors per document: 25). Microsoft Office Excel 2019 (Redmond, Washington, United States) was used to manage data. The correlation between the year and the number of articles was expressed by the linear correlation coefficient (R^2^).

## Results

**FIGURE 1:**
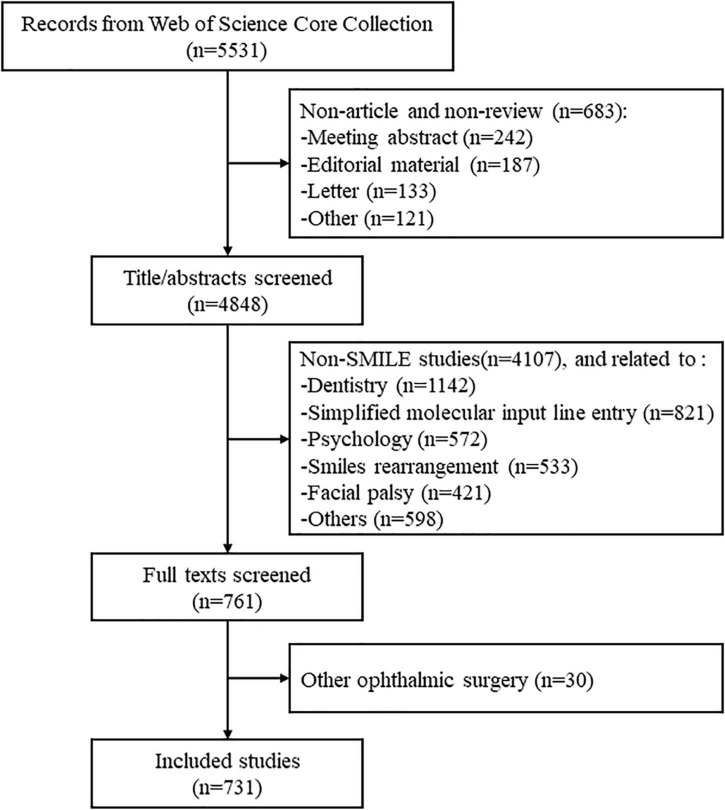
Results of literature search.

In total, 731 publications associated with SMILE in the WoSCC from 2011 to 2022 were identified (Figure 1), of which, 667 (91.24%) and 64 (8.76%) were indexed as “article” and “review,” respectively.

### Annual growth trend

**FIGURE 2:**
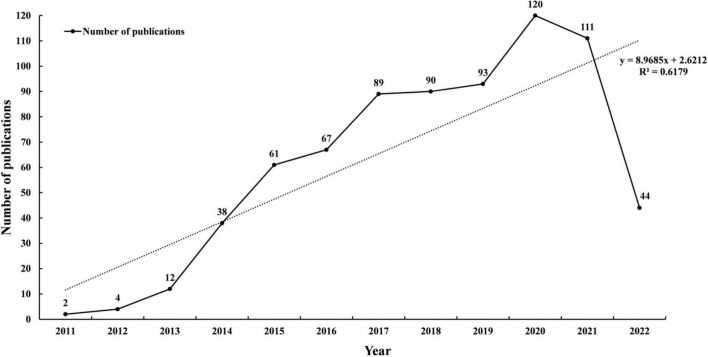
The number of publications per year (2011–2022).

Outputs of the annual publication with an upward trend are shown in Figure 2. There were two and four papers published in 2011 and 2012, respectively. The number of publications was 61 in 2015, which increased to more than 80 in 2017, and reached the highest in 2020 (*n* = 120). A significant correlation between the number of studies and the year of publication was found with a high coefficient of determination (*R*^2^ = 0.62).

### Countries/regions and institutions analysis

The top 10 countries or regions, and institutions among the 604 institutions in 45 countries are shown in Table 1, according to the number of publications.

**TABLE 1:** The top 10 most productive countries/regions and institution for SMILE research.

Rank	Country/region	*N* (%)	Rank	Institution	*N* (%)
1	China (Asia)	326 (44.60)	1	Fudan University	103 (14.09)
2	United States (North America)	140 (19.15)	2	Tianjin Medical University	51 (6.98)
3	Germany (Europe)	81 (12.37)	3	Sun Yat-sen University	43 (5.88)
4	India (Asia)	68 (9.30)	4	Singapore National Eye Centre	42 (5.75)
5	France (Europe)	58 (7.93)	5	Duke-NUS Medical School	39 (5.34)
6	United Kingdom (Europe)	58 (7.93)	6	Singapore Eye Research Institute	39 (5.34)
7	Singapore (Asia)	46 (6.29)	7	London Vision Clinic	37 (5.06)
8	South Korea (Asia)	39 (5.34)	8	Shanghai Research Center of Ophthalmology and Optometry	37 (5.06)
9	Spain (Europe)	36 (4.92)	9	Columbia University	35 (4.79)
10	Denmark (Europe)	32 (4.38)	10	Aarhus University Hospital	28 (3.83)

**FIGURE 3:**
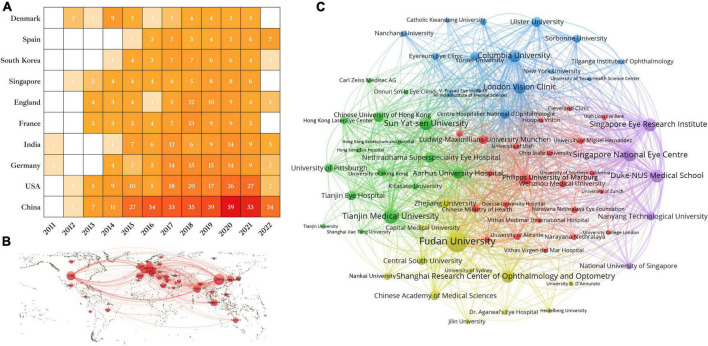
The annual output of publications in the top 10 countries/regions **(A)**, the network map of countries/regions **(B)**, and institutions **(C)** related to SMILE research.

A total of 45 countries/regions contributed to SMILE research. China published the highest number of papers (*n* = 326), followed by the United States (*n* = 140), Germany (*n* = 81), India (*n* = 68), and France (*n* = 58). As shown in Figure 3A, the annual output of most countries showed an upward trend. All countries/regions were used to construct a country/region network map (Figure 3B).

The top 10 institutions were distributed in five countries/regions, four of which were in China (Table 1). As Figure 3C shows, institutions (70/604, 11.59%) with six or more (T = 6) publications were used to construct the co-authorship network. The institutions were then divided into six clusters of different colors.

### Journal analysis

Seventy-seven scholarly journals published papers on SMILE research. Over 250 papers were published in the top two journals, both of which were published in the United States (Table 2). The *Journal of Refractive Surgery* published the most papers (*n* = 166), followed by the *Journal of Cataract and Refractive Surgery* (*n* = 98), and *BMC Ophthalmology* (*n* = 48). Among 1144 co-cited academic journals, five had more than 1000 citations, and all of them were from the United States. The *Journal of Refractive Surgery* had the most co-citations (*n* = 4427), followed by the *Journal of Cataract and Refractive Surgery* (*n* = 3928), *Investigative Ophthalmology & Visual Science* (*n* = 1252), *Cornea* (*n* = 1217), and *Ophthalmology* (*n* = 1155).

**TABLE 2:** The top 10 productive journals and co-cited journals of SMILE research.

Rank	Journal	N	IF2021^a^	Q^b^	Co-cited journal	Co-citation	IF2021	Q
1	Journal of Refractive Surgery (United States)	166	3.255	Q2	Journal of Refractive Surgery (United States)	4427	3.255	Q2
2	Journal of Cataract and Refractive Surgery (United States)	98	3.528	Q2	Journal of Cataract and Refractive Surgery (United States)	3928	3.528	Q2
3	BMC Ophthalmology (England)	48	2.090	Q3	Investigative Ophthalmology & Visual Science (United States)	1252	4.925	Q1
4	Cornea (United States)	42	3.152	Q2	Cornea (United States)	1217	3.152	Q2
5	Journal of Ophthalmology (United States)	29	1.974	Q4	Ophthalmology (United States)	1155	14.277	Q1
6	PLoS One (United States)	23	3.752	Q2	British Journal of Ophthalmology (England)	913	5.915	Q1
7	Indian Journal of Ophthalmology (India)	18	2.969	Q3	American Journal of Ophthalmology (United States)	774	5.488	Q1
8	International Journal of Ophthalmology (China)	18	1.647	Q4	Graefes Archive for Clinical and Experimental Ophthalmology (United States)	627	3.535	Q2
9	Graefes Archive for Clinical and Experimental Ophthalmology (United States)	17	3.535	Q2	PLoS One (United States)	527	3.752	Q2
10	International Ophthalmology (Netherlands)	17	2.029	Q3	Clinical Ophthalmology (England)	367	-	-

^a^lmpact factor of the journals that was provided by the 2021 Journal Citation Report. ^b^Quartile in category that was provided by the 2021 Journal Citation Reports.

**FIGURE 4:**
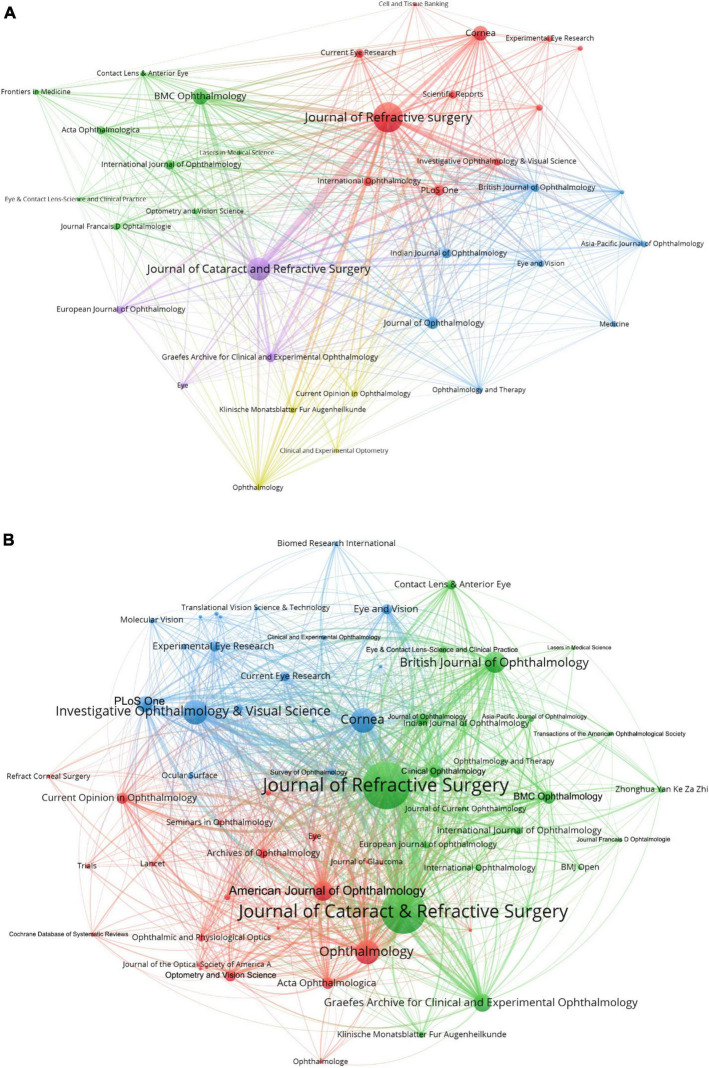
The network map of scholarly journals **(A)** and co-cited scholarly journals **(B)** for SMILE research.

Journals (36/77, 46.75%) with a publication number greater than or equal to three (T = 3) were used to construct the citation network map, which can be divided into five clusters with different colors (Figure 4A).

Journals (65/1144, 5.68%) with co-citations greater than or equal to 25 (T = 25) were used to construct the co-citation network (Figure 4B).

### Keywords analysis

**FIGURE 5:**
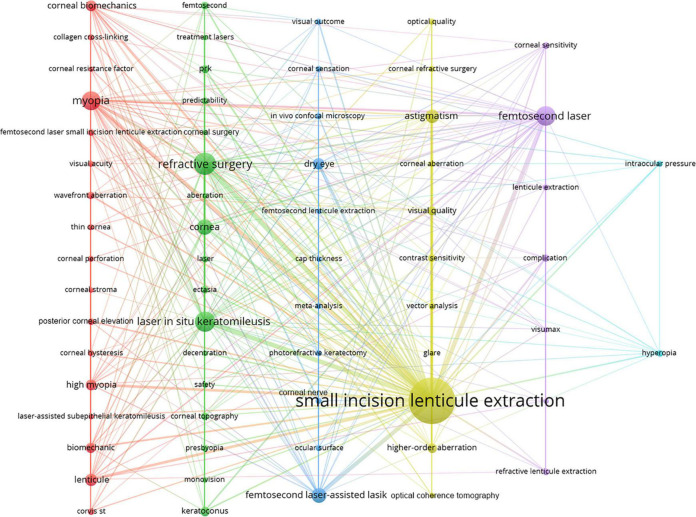
The network map of keywords for SMILE research.

A total of 686 hotspot keywords for SMILE research were extracted with a frequency of occurrence of 1720. Subsequently, 65 keywords that appeared more than four times were included and classified into six clusters on the map (Figure 5), including cluster 1 (biomechanics, collagen cross-linking, in red), cluster 2 (ectasia and corneal topography, in green), cluster 3 (dry eye and corneal sensation, in blue), cluster 4 (visual quality, glare, and astigmatism, in yellow), cluster 5 (complication and femtosecond laser, in purple), and cluster 6 (hyperopia and intraocular pressure, in bright blue).

### Reference analysis

The top 10 co-cited references in SMILE research are listed in Table 3. Each reference was co-cited at least 93 times.

**TABLE 3:** The top 10 co-cited references in SMILE research.

Rank	References	N
1	Sekundo et al. (3)	371
2	Shah et al. (4)	293
3	Ivarsen et al. (5)	184
4	Vestergaard et al. (6)	140
5	Reinstein et al. (7)	131
6	Sekundo et al. (2)	127
7	Sekundo et al. (8)	113
8	Ganesh and Gupta (9)	109
9	Hjortdal et al. (10)	103
10	Wu et al. (11)	93

### Citation bursts

The top 25 citation bursts pertaining to the development of SMILE were identified from 2011 to 2022 (Table 4). The increasing number of citations of these papers in a certain period indicated rapid dissemination. Among them, the first citation burst appeared in 2011, while the last eight citation bursts began after 2020.

**TABLE 4:** The top 25 references with the strongest citation bursts in the co-citation network.

References	Strength	Begin	End	2011–2022
Blum et al. (12)	21.06	2011	2015	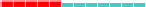
Sekundo et al. (3)	49.3	2012	2016	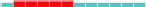
Shah et al. (4)	39.41	2012	2016	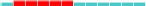
Vestergaard et al. (6)	17.2	2012	2017	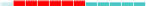
Ang et al. (13)	10.54	2012	2015	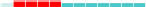
Hjortdal et al. (10)	12.84	2013	2017	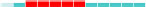
Riau et al. (14)	12.45	2013	2016	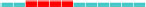
Vestergaard et al. (15)	8.1	2013	2014	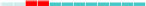
Kamiya et al. (16)	7.01	2013	2015	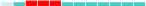
Wei et al. (17)	6.87	2013	2015	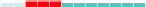
Tay et al. (18)	6.31	2013	2015	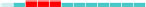
Shah et al. (4)	8.99	2014	2016	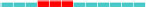
Gertnere et al. (19)	6.89	2014	2015	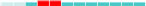
Reinstein et al. (7)	6.26	2014	2017	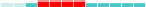
Shetty et al. (20)	8.64	2019	2022	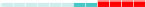
Ganesh et al. (21)	7.58	2019	2022	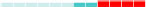
Zhang et al. (22)	7.48	2019	2022	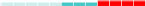
Blum et al. (23)	15.41	2020	2022	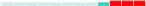
Han et al. (24)	10.41	2020	2022	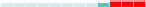
Kim et al. (1)	10.09	2020	2022	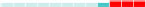
Li et al. (25)	8.64	2020	2022	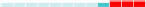
Titiyal et al. (26)	8.48	2020	2022	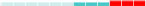
Han et al. (27)	8.41	2020	2022	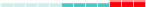
Wang et al. (28)	8.11	2020	2022	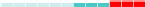
Daingaard et al. (29)	6.71	2020	2022	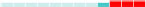

The red line means strong citation burst timeline, while the blue line means infrequent citation timeline.

## Discussion

Bibliometrics is helpful for understanding the evolutionary process of a discipline. Through keyword analysis, we found that the research keywords for SMILE surgery can be classified into six clusters: femtosecond laser technology, dry eye, biomechanics, visual quality, complications, and hyperopia. These keywords reflect the main concerns of clinicians and researchers regarding the development of SMILE surgery. Thus, we have discussed the development of SMILE in the following order: basic information and six clusters.

### Basic information

Studies on SMILE surgery peaked in 2020. The number of articles published in various countries is mostly on the rise, indicating the rapid development of SMILE. Among them, China has the largest number of papers. This may be due to an early start of SMILE surgery in China. Currently, SMILE has become the most common corneal surgical method for myopia correction in China, and more than three million SMILE surgeries have been completed in China. Therefore, it is not surprising to find that Chinese research institutions occupy the top four of 10 seats with good international collaboration. The United States is potent enough to mention here, as SMILE surgery was approved by the United States Food and Drug Administration (FDA) in 2016 (30). In addition, as shown in Figure 3B, a tight collaboration was observed between the United States and China. The *Journal of Refractive Surgery* and *Journal of Cataract and Refractive Surgery* ranked first and second in the lists of the most productive and co-cited journals, respectively.

### Femtosecond laser technology

Owing to the development of femtosecond lasers, corneal laser surgery has entered the femtosecond era. The femtosecond laser replaced the keratome, which was the preferred tool for laser-assisted *in situ* keratomileusis (LASIK) flap making. Thereafter, the FLEx (2) and SMILE surgery (3, 4) came into being, followed by gradual development. The most cited literature in SMILE-related research was published between 2008 and 2014 (Table 3). During this period, the surgical technique for SMILE was still in its infancy, and the most important discussion was associated with the predictability and safety of SMILE surgery since they were the focuses of most of the top 10 co-cited studies (3, 4, 6, 8–10, 31).

Furthermore, predictability has remained a hotspot. Initial research focused on early postoperative predictability, but current researchers are more concerned about long-term predictability since five of the 11 top references with the strongest citation bursts in 2022 were focused on this aspect (Table 4). Several studies have shown exciting long-term predictivity, stability, safety, and high patient satisfaction (24, 32). This provides clinicians with confidence in SMILE surgery. However, factors related to corneal regression and comparisons between SMILE and other refractive surgeries are worth investigating.

### Biomechanics

Since the incision of SMILE is small, another interest is whether fewer changes in corneal biomechanics occur during SMILE surgery. Researchers have compared biomechanical results of SMILE and LASIK using dynamic Scheimpflug imaging (Corvis ST) (20, 33), Ocular Response Analyzer (ORA) (34), mathematical analyses (7, 35), as well as meta-analysis (36), and demonstrated that SMILE seems to be superior to LASIK in terms of preserving corneal biomechanics. However, common problems of corneal refractive surgery, such as refractive regression and postoperative corneal ectasia, still cannot be avoided in SMILE surgery. Nineteen cases of corneal ectasia after SMILE surgery have been reported since 2017, although the previous tomography findings were not normal in some of these cases. Based on the literature review of ectasia, the incidence of post-refractive ectasia in eyes without identifiable preoperative risk factors is 20 per 100,000 eyes for photorefractive keratectomy (PRK), 90 per 100,000 eyes for LASIK, and 11 per 100,000 eyes for SMILE (37). In addition, it is believed that the combination of Corvis ST and Pentacam can fill the void in preoperative risk prediction and early diagnosis of corneal ectasia and keratoconus; however, the data output by Corvis ST still warrants further discussion.

### Visual quality

The visual quality of SMILE surgery has been widely studied. Aberration is the most commonly used method for evaluating visual quality. Many studies have shown that a smaller spherical aberration was induced by SMILE compared to LASIK (24, 38, 39), which might be due to the larger optical zone after SMILE (24). With the emergence of new detection methods, results of the visual quality of SMILE surgery using methods such as optical quality assessment system (OQAS) (40, 41), Oculus Cataract Quantifier (C-Quant) (42), disk halo sizes (43), and corneal densitometry (44) have been reported successively. The overall trend indicates that SMILE surgery has no significant impact on visual quality three months postoperatively. No significant difference in corneal transparency has been shown in the first postoperative week (44).

Astigmatism is another important research topic. Unlike LASIK, in which an infrared-guided pupil tracking system is used during the surgical process, SMILE mainly depends on the surgeon’s judgment. Consequently, the comparison of astigmatism vector analysis between the two surgeries has attracted much attention. Although there is no unified conclusion at present, it is reported that there is slight inferiority and more under-correction during SMILE than during LASIK when treating low-to-moderate astigmatism, and a comparable rate of under-correction when treating high astigmatism (45, 46). Comparing outcomes of astigmatism correction of SMILE surgery with those of other surgical methods, as well as different types of astigmatism correction, need to be studied. In addition, software that enhances eye tracking or cyclotorsion compensation is also being developed and will soon be available (1).

### Complications

Complications of SMILE are a constant concern, especially postoperative complications such as corneal ectasia. Other common postoperative complications include dry eyes and diffuse lamellar keratitis (47).

Initially, intraoperative complications gained more attention than postoperative ones, as a certain learning curve is required for surgeons performing SMILE. In 2014, Ivarsen et al. (5) published a study based on clinical results and surgical complications of more than 1,500 SMILE operations. This article is also the third in the top co-cited references in SMILE-related research and the first clinical practice result for SMILE with a large sample. In this study, tearing at the incision (114/1800) and difficulties in lenticule separation (34/1800) are common causes of intraoperative complications. Other common intraoperative complications include suction loss, opaque bubble layer (OBL), and black spots (47). In the initial period of the learning curve for SMILE, some patients may experience delayed vision recovery, although the phenomenon may be improved by lowering the femtosecond laser energy and advancing the surgical experience (48). In general, SMILE is associated with rapid visual recovery, and most patients can achieve 20/20 visual acuity within one day after surgery according to our and other surgeons’ experience (49).

### Dry eye

The main highlight of SMILE is the characteristic small incision. Compared to the approximately 20 mm incision in LASIK, a small incision design guarantees a reduction in flap-related complications, such as flap loss and flap displacement. In addition, it also reduces damage to the corneal nerves. Compared with LASIK, the recovery time of corneal sensation and dry eye symptoms after SMILE has been proved to be shortened through clinical research and experimental research (50, 51).

### Hyperopia

Treatment of hyperopia with SMILE is not easy. The result of the earliest attempt using FLEx was not satisfactory. By enlarging the transition zone in SMILE, its stability is improved, and the refractive outcomes are similar to those of LASIK (52, 53). At present, global clinical observation of hyperopia SMILE has led to preliminary results, and it is believed that hyperopia SMILE will be progressing in the next few years (54).

For correction of hyperopia, an important and attractive surgical correction is the transplantation of lenticules obtained from the SMILE procedure (55). Lenticule keratophakia and epikeratophakia are reversible in SMILE, and the visual quality offers unique advantages (56, 57). Moreover, the discarded tissue was also reused as a bio-scaffold for stromal engineering, and an ocular drug delivery system of active molecules (58, 59).

## Conclusion

The number of literature has been growing rapidly in the past 12 years. Our study provides a deep insight into publications on SMILE for researchers and clinicians with bibliometric analysis for the first time.

## Data availability statement

The raw data supporting the conclusions of this article will be made available by the authors, without undue reservation.

## Author contributions

TH and XZ: conceptualization. LZ: data curation. TH and LZ: writing original draft preparation. All authors: reviewing and editing.

## References

[1] Kim TI Alio Del Barrio JL Wilkins M Cochener B Ang M. Refractive surgery. *Lancet.* (2019) 393:2085–98. 10.1016/S0140-6736(18)33209-4 31106754

[2] Sekundo W Kunert K Russmann C Gille A Bissmann W Stobrawa G First efficacy and safety study of femtosecond lenticule extraction for the correction of myopia: six-month results. *J Cataract Refract Surg.* (2008) 34:1513–20. 10.1016/j.jcrs.2008.05.033 18721712

[3] Sekundo W Kunert KS Blum M. Small incision corneal refractive surgery using the small incision lenticule extraction (SMILE) procedure for the correction of myopia and myopic astigmatism: results of a 6 month prospective study. *Br J Ophthalmol.* (2011) 95:335–9. 10.1136/bjo.2009.174284 20601657

[4] Shah R Shah S Sengupta S. Results of small incision lenticule extraction: all-in-one femtosecond laser refractive surgery. *J Cataract Refract Surg.* (2011) 37:127–37. 10.1016/j.jcrs.2010.07.033 21183108

[5] Ivarsen A Asp S Hjortdal J. Safety and complications of more than 1500 small-incision lenticule extraction procedures. *Ophthalmology.* (2014) 121:822–8. 10.1016/j.ophtha.2013.11.006 24365175

[6] Vestergaard A Ivarsen AR Asp S Hjortdal JO. Small-incision lenticule extraction for moderate to high myopia: Predictability, safety, and patient satisfaction. *J Cataract Refract Surg.* (2012) 38:2003–10. 10.1016/j.jcrs.2012.07.021 22981612

[7] Reinstein DZ Archer TJ Randleman JB. Mathematical model to compare the relative tensile strength of the cornea after PRK, LASIK, and small incision lenticule extraction. *J Refract Surg.* (2013) 29:454–60. 10.3928/1081597X-20130617-03 23820227

[8] Sekundo W Gertnere J Bertelmann T Solomatin I. One-year refractive results, contrast sensitivity, high-order aberrations and complications after myopic small-incision lenticule extraction (ReLEx SMILE). *Graefes Arch Clin Exp Ophthalmol.* (2014) 252:837–43. 10.1007/s00417-014-2608-4 24647595

[9] Ganesh S Gupta R. Comparison of visual and refractive outcomes following femtosecond laser– assisted lasik with smile in patients with myopia or myopic astigmatism. *J Refract Surg.* (2014) 30:590–6. 25250415 10.3928/1081597X-20140814-02

[10] Hjortdal JO Vestergaard AH Ivarsen A Ragunathan S Asp S. Predictors for the outcome of small-incision lenticule extraction for Myopia. *J Refract Surg.* (2012) 28:865–71. 10.3928/1081597X-20121115-01 23231737

[11] Wu D Wang Y Zhang L Wei SS Tang V. Corneal biomechanical effects: small incision lenticule extraction versus femtosecond laser-assisted laser in situ keratomileusis. *J Cataract Refract Surg.* (2014) 40:954–62. 10.1016/j.jcrs.2013.07.056 24751146

[12] Blum M Kunert K Schröder M Sekundo W. Femtosecond lenticule extraction for the correction of myopia: preliminary 6-month results. *Graefes Arch Clin Exp Ophthalmol.* (2010) 248:1019–27. 10.1007/s00417-009-1293-1 20130899

[13] Ang M Chaurasia SS Angunawela RI Poh R Riau A Tan D Femtosecond lenticule extraction (FLEx): clinical results, interface evaluation, and intraocular pressure variation. *Invest Ophthalmol Vis Sci.* (2012) 53:1414-21. 10.1167/iovs.11-8808. 22323464

[14] Riau AK Angunawela RI Chaurasia SS Lee WS Tan DT Mehta JS. Early corneal wound healing and inflammatory responses after refractive lenticule extraction (ReLEx). *Invest Ophthalmol Vis Sci.* (2011) 52:6213–21. 10.1167/iovs.11-7439. 21666235

[15] Vestergaard A Ivarsen A Asp S. Hjortdal JO. Femtosecond (FS) laser vision correction procedure for moderate to high myopia: a prospective study of ReLEx^®^ flex and comparison with a retrospective study of FS-laser in situ keratomileusis. *Acta Ophthalmologica.* (2012) 91:355–62. 10.1111/j.1755-3768.2012.02406.x. 22512839

[16] Kamiya K Igarashi A Ishii R Sato N Nishimoto H Shimizu K. Early clinical outcomes, including efficacy and endothelial cell loss, of refractive lenticule extraction using a 500 kHz femtosecond laser to correct myopia. *J Cataract Refract Surg.* (2012) 38:1996–2002. 10.1016/j.jcrs.2012.06.052. 22981613

[17] Wei S Wang Y. Comparison of corneal sensitivity between FS-LASIK and femtosecond lenticule extraction (ReLEx flex) or small-incision lenticule extraction (ReLEx smile) for myopic eyes. *Graefes Arch Clin Exp Ophthalmol.* (2013) 251:1645–54. 10.1007/s00417-013-2272-0. 23389552

[18] Tay E Li X Chan C Tan DT Mehta JS. Refractive lenticule extraction flap and stromal bed morphology assessment with anterior segment optical coherence tomography. *J Cataract Refract Surg.* (2012) 38:1544–51. 10.1016/j.jcrs.2012.05.030. 22906441

[19] Gertnere J Solomatin I Sekundo W. Refractive lenticule extraction (ReLEx flex) and wavefront-optimized Femto-LASIK: comparison of contrast sensitivity and high-order aberrations at 1 year. *Graefes Arch Clin Exp Ophthalmol.* (2013) 251:1437–42. 10.1007/s00417-012-2220-4. 23212799

[20] Shetty R Francis M Shroff R Pahuja N Khamar P Girrish M Corneal biomechanical changes and tissue remodeling after SMILE and LASIK. *Invest Ophthalmol Vis Sci.* (2017) 58:5703–12. 10.1167/iovs.17-22864 29101408

[21] Ganesh S Brar S Pawar A. Results of intraoperative manual cyclotorsion compensation for myopic astigmatism in patients undergoing small incision lenticule extraction (SMILE). *J Refract Surg.* (2017) 33:506–12. 10.3928/1081597X-20170328-01. 28787514

[22] Zhang Y Shen Q Jia Y Zhou D Zhou J. Clinical outcomes of SMILE and FS-LASIK used to treat myopia: a meta-analysis. *J Refract Surg.* (2016) 32:256–65. 10.3928/1081597X-20151111-06. 27070233

[23] Blum M Lauer AS Kunert KS Sekundo W. 10-year results of small incision lenticule extraction. *J Refract Surg.* (2019) 35:618–23. 10.3928/1081597X-20190826-02. 31610002

[24] Han T Xu Y Han X Zeng L Shang J Chen X Three-year outcomes of small incision lenticule extraction (SMILE) and femtosecond laser-assisted laser in situ keratomileusis (FS-LASIK) for myopia and myopic astigmatism. *Br J Ophthalmol.* (2019) 103:565–8. 10.1136/bjophthalmol-2018-312140 30061116 PMC6691872

[25] Li M Li M Chen Y Miao H Yang D Ni K Zhou X. Five-year results of small incision lenticule extraction (SMILE) and femtosecond laser LASIK (FS-LASIK) for myopia. *Acta Ophthalmol.* (2019) 97:e373–80. 10.1111/aos.14017. 30632671

[26] Titiyal JS Kaur M Rathi A Falera R Chaniyara M Sharma N. Learning curve of small incision lenticule extraction: challenges and complications. *Cornea.* (2017) 36:1377–82. 10.1097/ICO.0000000000001323. 28799958

[27] Han T Zheng K Chen Y Gao Y He L Zhou X. Four-year observation of predictability and stability of small incision lenticule extraction. *BMC Ophthalmol.* (2016) 16:149. 10.1186/s12886-016-0331-0. 27577086 PMC5006606

[28] Wang Y Ma J Zhang J Dou R Zhang H Li L Incidence and management of intraoperative complications during small-incision lenticule extraction in 3004 cases. *J Cataract Refract Surg.* (2017) 43:796–802. 10.1016/j.jcrs.2017.03.039. 28732614

[29] Damgaard IB Ang M Mahmoud AM Farook M Roberts CJ Mehta JS. Functional optical zone and centration following SMILE and LASIK: a prospective, randomized, contralateral eye study. *J Refract Surg.* (2019) 35:230–37. 10.3928/1081597X-20190313-01. 30984980

[30] Food and Drug Administration [FDA]. *The U.S. is Potent Enough To Mention Here, as Smile Surgery Was 200*. Silver Spring, MA: FDA (2016).

[31] Kamiya K Shimizu K Igarashi A Kobashi H. Visual and refractive outcomes of femtosecond lenticule extraction and small-incision lenticule extraction for myopia. *Am J Ophthalmol.* (2014) 157:128.e–34.e. 10.1016/j.ajo.2013.08.011 24112634

[32] Ağca A Tülü B Yaşa D Yıldırım Y Yıldız BK Demirok A. Long-term (5 years) follow-up of small-incision lenticule extraction in mild-to-moderate myopia. *J Cataract Refract Surg.* (2019) 45:421–6. 10.1016/j.jcrs.2018.11.010 30709628

[33] Shen Y Chen Z Knorz MC Li M Zhao J Zhou X. Comparison of corneal deformation parameters after SMILE, LASEK, and femtosecond laser-assisted LASIK. *J Refract Surg.* (2014) 30:310–8. 10.3928/1081597x-20140422-01 24904933

[34] Osman IM Helaly HA Abdalla M Shousha MA. . Corneal biomechanical changes in eyes with small incision lenticule extraction and laser assisted in situ keratomileusis. *BMC Ophthalmol.* (2016) 16:123. 10.1186/s12886-016-0304-3 27457241 PMC4960872

[35] Seven I Vahdati A Pedersen IB Vestergaard A Hjortdal J Roberts CJ Contralateral eye comparison of SMILE and flap-based corneal refractive surgery: computational analysis of biomechanical impact. *J Refract Surg.* (2017) 33:444–53. 10.3928/1081597X-20170504-01 28681903 PMC6041122

[36] Guo H Hosseini-Moghaddam SM Hodge W. Corneal biomechanical properties after SMILE versus FLEX, LASIK, LASEK, or PRK: a systematic review and meta-analysis. *BMC Ophthalmol.* (2019) 19:167. 10.1186/s12886-019-1165-3 31370817 PMC6676534

[37] Moshirfar M Tukan AN Bundogji N Liu HY McCabe SE Ronquillo YC Ectasia after corneal refractive surgery: a systematic review. *Ophthalmol Ther.* (2021) 10:753–76. 10.1007/s40123-021-00383-w 34417707 PMC8589911

[38] Gyldenkerne A Ivarsen A Hjortdal JO. Comparison of corneal shape changes and aberrations induced By FS-LASIK and SMILE for myopia. *J Refract Surg.* (2015) 31:223–9. 10.3928/1081597X-20150303-01 25751842

[39] Yao L Zhang M Wang D Zhao Q Wang S Bai H. Small incision lenticule. extraction (SMILE) and laser in situ keratomileusis (LASIK) used to treat myopia and myopic astigmatism: a systematic review and meta-analysis of randomized clinical trials. *Semin Ophthalmol.* (2022) 1–11. 10.1080/08820538.2022.2107399 [Epub ahead of print]. 35912896

[40] Miao H He L Shen Y Li M Yu Y Zhou X. Optical quality and intraocular scattering after femtosecond laser small incision lenticule extraction. *J Refract Surg.* (2014) 30:296–302. 10.3928/1081597X-20140415-02 24893354

[41] Kamiya K Shimizu K Igarashi A Kobashi H. Effect of femtosecond laser setting on visual performance after small-incision lenticule extraction for myopia. *Br J Ophthalmol.* (2015) 99:1381–7. 10.1136/bjophthalmol-2015-306717 25855501

[42] Luo J Yao P Li M Xu G Zhao J Tian M Quantitative analysis of microdistortions in bowman’s layer using optical coherence tomography after SMILE among different myopic corrections. *J Refract Surg.* (2015) 31:104–9. 10.3928/1081597X-20150122-05. 25735043

[43] Han T Zhao F Chen X Miao H Chen Z Zhou X. Evaluation of disk halo size after small incision lenticule extraction (SMILE). *Graefes Arch Clin Exp Ophthalmol.* (2019) 257:2789–93. 10.1007/s00417-019-04481-1. 31664518

[44] Han T Zhao J Shen Y Chen Y Tian M Zhou X. A three-year observation of corneal backscatter after small incision lenticule extraction (SMILE). *J Refract Surg.* (2017) 33:377–82. 10.3928/1081597X-20170420-01. 28586497

[45] Chan TC Ng AL Cheng GP Wang Z Ye C Woo VC Vector analysis of astigmatic correction after small-incision lenticule extraction and femtosecond-assisted LASIK for low to moderate myopic astigmatism. *Br J Ophthalmol.* (2016) 100:553–9. 10.1136/bjophthalmol-2015-307238 26206791

[46] Chow SSW Chow LLW Lee CZ Chan TCY. Astigmatism correction using SMILE. *Asia Pac J Ophthalmol (Phila).* (2019) 8:391–6. 10.1097/01.APO.0000580140.74826.f5 31490198 PMC6784860

[47] Asif MI Bafna RK Mehta JS Reddy J Titiyal JS Maharana PK Complications of small incision lenticule extraction. *Indian J Ophthalmol.* (2020) 68:2711–22. 10.4103/ijo.IJO_3258_20 33229647 PMC7856979

[48] Ji YW Kim M Kang DSY Reinstein DZ Archer TJ Choi JY Lower laser energy levels lead to better visual recovery after small-incision lenticule extraction: prospective randomized clinical trial. *Am J Ophthalmol.* (2017) 179:159–70. 10.1016/j.ajo.2017.05.005 28499707

[49] Ahmed AA Hatch KM. Advantages of small incision lenticule extraction (SMILE) for mass eye and ear special issue. *Semin Ophthalmol.* (2020) 35:224–31. 10.1080/08820538.2020.1807028 32892680

[50] Mohamed-Noriega K Riau AK Lwin NC Chaurasia SS Tan DT Mehta JS. Early corneal nerve damage and recovery following small incision lenticule extraction (SMILE) and laser in situ keratomileusis (LASIK). *Invest Ophthalmol Vis Sci.* (2014) 55:1823–34. 10.1167/iovs.13-13324 24569584

[51] Denoyer A Landman E Trinh L Faure JF Auclin F Baudouin C. Dry eye disease after refractive surgery: comparative outcomes of small incision lenticule extraction versus LASIK. *Ophthalmology.* (2015) 122:669–76. 10.1016/j.ophtha.2014.10.004 25458707

[52] Pradhan KR Reinstein DZ Carp GI Archer TJ Dhungana P. Small incision lenticule extraction (SMILE) for hyperopia: 12-month refractive and visual outcomes. *J Refract Surg.* (2019) 35:442–50. 10.3928/1081597X-20190529-01 31298724

[53] Moshirfar M Bruner CD Skanchy DF Shah T. Hyperopic small-incision lenticule extraction. *Curr Opin Ophthalmol.* (2019) 30:229–35. 10.1097/ICU.0000000000000580 31033739

[54] Ang M Gatinel D Reinstein DZ Mertens E Alio Del Barrio JL Alió JL. Refractive surgery beyond 2020. *Eye (Lond).* (2021) 35:362–82. 10.1038/s41433-020-1096-5 32709958 PMC8027012

[55] Sun L Yao P Li M Shen Y Zhao J Zhou X. The safety and predictability of implanting autologous lenticule obtained by SMILE for hyperopia. *J Refract Surg.* (2015) 31:374–9. 10.3928/1081597X-20150521-03 26046703

[56] Liu YC Wen J Teo EPW Williams GP Lwin NC Mehta JS. Higher-order-aberrations following hyperopia treatment: small incision lenticule extraction, laser-assisted in situ keratomileusis and lenticule implantation. *Transl Vis Sci Technol.* (2018) 7:15. 10.1167/tvst.7.2.15 29616154 PMC5879992

[57] Riau AK Liu YC Yam GHF Mehta JS. Stromal keratophakia: corneal inlay implantation. *Prog Retin Eye Res.* (2020) 75:100780. 10.1016/j.preteyeres.2019.100780 31493488

[58] Mastropasqua L Nubile M Acerra G Detta N Pelusi L Lanzini M Bioengineered human stromal lenticule for recombinant human nerve growth factor release: a potential biocompatible ocular drug delivery system. *Front Bioeng Biotechnol.* (2022) 10:887414. 10.3389/fbioe.2022.887414 35813999 PMC9260024

[59] Santra M Liu YC Jhanji V Yam GH. Human SMILE-derived stromal lenticule scaffold for regenerative therapy: review and perspectives. *Int J Mol Sci.* (2022) 23:79670. 10.3390/ijms23147967 35887309 PMC9315730

